# A Case of Myxoma-Like Degeneration in a Patient With Marfan Syndrome Mimicking Vegetation of Infective Endocarditis

**DOI:** 10.7759/cureus.114210

**Published:** 2026-08-09

**Authors:** Koki Sugiyama, Takayuki Ishige, Hayato Yasui, Hiroya Yokohari, Hiroyuki Takao, Yoichi Iwamoto, Hirotaka Ishido, Satoshi Masutani

**Affiliations:** 1 Department of Pediatrics, Saitama Medical Center, Saitama Medical University, Kawagoe, JPN

**Keywords:** diagnosis, echocardiography, genetic testing, missense mutation in fbn1, mitral valve prolapse

## Abstract

Infective endocarditis (IE) is often considered first in the differential diagnosis when fever, a heart murmur, and valvular vegetation are present. IE caused by *Mycoplasma pneumoniae *has rarely been reported. We report a case in which IE was initially suspected based on the presence of fever, a heart murmur, and an IE vegetation-like structure on the anterior leaflet of the mitral valve identified by echocardiography following admission for pneumonia caused by *M. pneumoniae*. However, IE was ultimately excluded, and the vegetation-like structure was determined to represent myxoma-like degeneration associated with Marfan syndrome. A seven-year-old girl with no notable family history and no characteristic physical findings presented with fever and pneumonia, and polymerase chain reaction testing was positive for *M. pneumoniae*. Echocardiography revealed an IE vegetation-like structure on the anterior mitral leaflet with leaflet prolapse, moderate mitral regurgitation, and enlargement of the aortic sinus of Valsalva. IE, due to *M. pneumoniae*, was suspected, and antimicrobial therapy was initiated. The patient's fever and pneumonia improved; however, the vegetation-like structure on the mitral valve remained unchanged. Multiple blood cultures were negative. Given suspicion for an underlying connective tissue disorder, genetic testing was performed and identified a missense mutation in FBN1, confirming Marfan syndrome. Because it is difficult to distinguish IE vegetation from myxoma-like degeneration associated with Marfan syndrome using echocardiography alone, recognition of this entity in patients with Marfan syndrome may improve diagnostic accuracy and prevent misdiagnosis.

## Introduction

Infective endocarditis (IE) should be considered in the differential diagnosis in patients presenting with fever, a heart murmur, and valvular vegetation. Diagnosis can be challenging, and the modified Duke criteria are widely used. The two major criteria include positive blood cultures and echocardiographic findings, whereas the five minor criteria include a history of heart disease, fever, vascular phenomena, immunologic phenomena, and microbiological evidence. The diagnosis of IE is based on a combination of these criteria [1]. Pediatric IE is rare, with an estimated incidence of less than one case per 100,000 children per year, and approximately 50-70% of cases occur in patients with underlying congenital heart disease [2]. In pediatric patients, careful evaluation of underlying conditions, such as congenital heart disease or the presence of central venous catheters, is particularly important. Common causative organisms include *Staphylococcus aureus*, *Streptococcus viridans*, and *Enterococcus* species [3].

*Mycoplasma pneumoniae*-associated IE is exceedingly uncommon. Only a few cases have been described in the literature. One report described a healthy adult with vegetation detected on echocardiography and positive serologic testing for *M. pneumoniae*; following doxycycline therapy, the vegetation resolved within two weeks [4]. Another case involved a 15-year-old boy with no prior medical history in whom vegetation was identified on echocardiography, and *M. pneumoniae *was successfully cultured. Treatment with intravenous clarithromycin, followed by oral levofloxacin for six months, resulted in a reduction in vegetation size [5].

Here, we report a pediatric case in which IE was initially suspected following admission for pneumonia caused by *M. pneumoniae* based on clinical findings and echocardiography. However, the final diagnosis was myxoma-like degeneration associated with Marfan syndrome rather than IE.

## Case presentation

A seven-year-old girl with no notable family history was admitted for pneumonia caused by *M. pneumoniae*, diagnosed using a FilmArray® respiratory panel multiplex polymerase chain reaction assay (BioFire Diagnostics) and chest radiography. Her height was 126 cm (+0.94 SD), and her weight was 21.8 kg (-0.43 SD) at admission, with standard deviation scores calculated using the Excel-based clinical tools for growth evaluation of children [6]. During hospitalization, a heart murmur was detected, and echocardiography revealed a vegetation-like structure suggestive of IE at the anterior leaflet of the mitral valve, accompanied by leaflet prolapse and moderate mitral regurgitation. Based on these findings, IE was suspected, and the patient was referred to our institution for further evaluation and management.

On admission, her body temperature was 37.2°C, with a heart rate of 102 beats/min, blood pressure of 91/71 mmHg, and oxygen saturation of 96% on room air. A grade II/VI systolic regurgitant murmur was auscultated at the cardiac apex. Laboratory findings are summarized in Table 1; some reference ranges were based on the pediatric reference ranges for laboratory tests provided by the National Center for Child Health and Development [7].

**Table 1:** Laboratory findings on admission Reference ranges marked with an asterisk (*) were based on the pediatric reference ranges for laboratory tests provided by the National Center for Child Health and Development [7]. APTT: activated partial thromboplastin time

Parameter	Value	Unit	Reference range
Total protein	6.5	g/dL	6.2-7.7*
Albumin	3.5	g/dL	3.6-4.7*
Creatine kinase	409	U/L	46-230*
Aspartate aminotransferase	37	U/L	24-38*
Alanine aminotransferase	15	U/L	9-27*
Lactate dehydrogenase	375	U/L	175-320*
Total bilirubin	0.5	mg/dL	0.3-0.9*
Blood urea nitrogen	6	mg/dL	6.6-19.6*
Creatinine	0.44	mg/dL	0.25-0.48*
Sodium	140	mEq/L	137-144*
Potassium	4.2	mEq/L	3.6-4.7*
Calcium	9.4	mg/dL	8.7-10.2*
C-reactive protein	6.82	mg/dL	0.00-0.14
APTT	46.6	sec	24.1-31.7
Prothrombin time	10.2	sec	-
Prothrombin time – international normalized ratio	1.05	-	-
Fibrinogen	443	mg/dL	193-412
D-dimer	2.63	µg/mL	0.00-0.99
White blood cell	7.1	×10³/µL	4.1-15.0*
Red blood cell	4.39	×10⁶/µL	4.1-5.2*
Hemoglobin	12.6	g/dL	11.5-14.4*
Hematocrit	36.7	%	34.5-43.0*
Platelet	240	×10³/µL	180-510*
Troponin I	10.0	pg/mL	0.0-26.2
Brain natriuretic peptide	5.8	pg/mL	0.0-18.4

**Figure 1:**
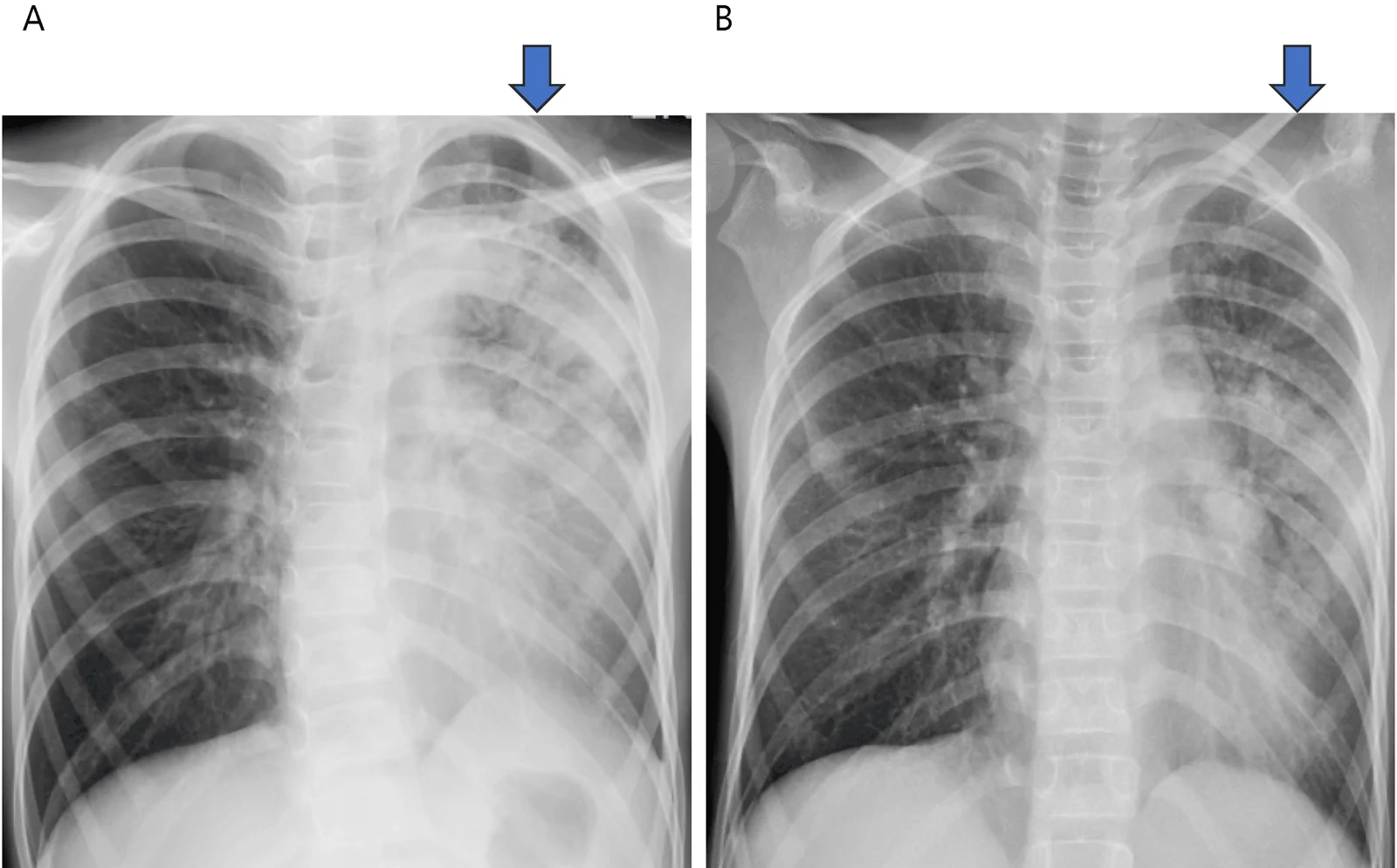
Chest radiography (A) Extensive ground-glass opacities are observed in the left lung field on admission. (B) Ground-glass opacity in the left lung field has improved significantly.

It revealed a white blood cell count of 7,100/μL (neutrophils: 56.3%; lymphocytes: 28.2%) and an elevated C-reactive protein level of 6.82 mg/dL. Chest radiography demonstrated extensive bilateral ground-glass opacities (Figure 1A).

**Figure 2:**
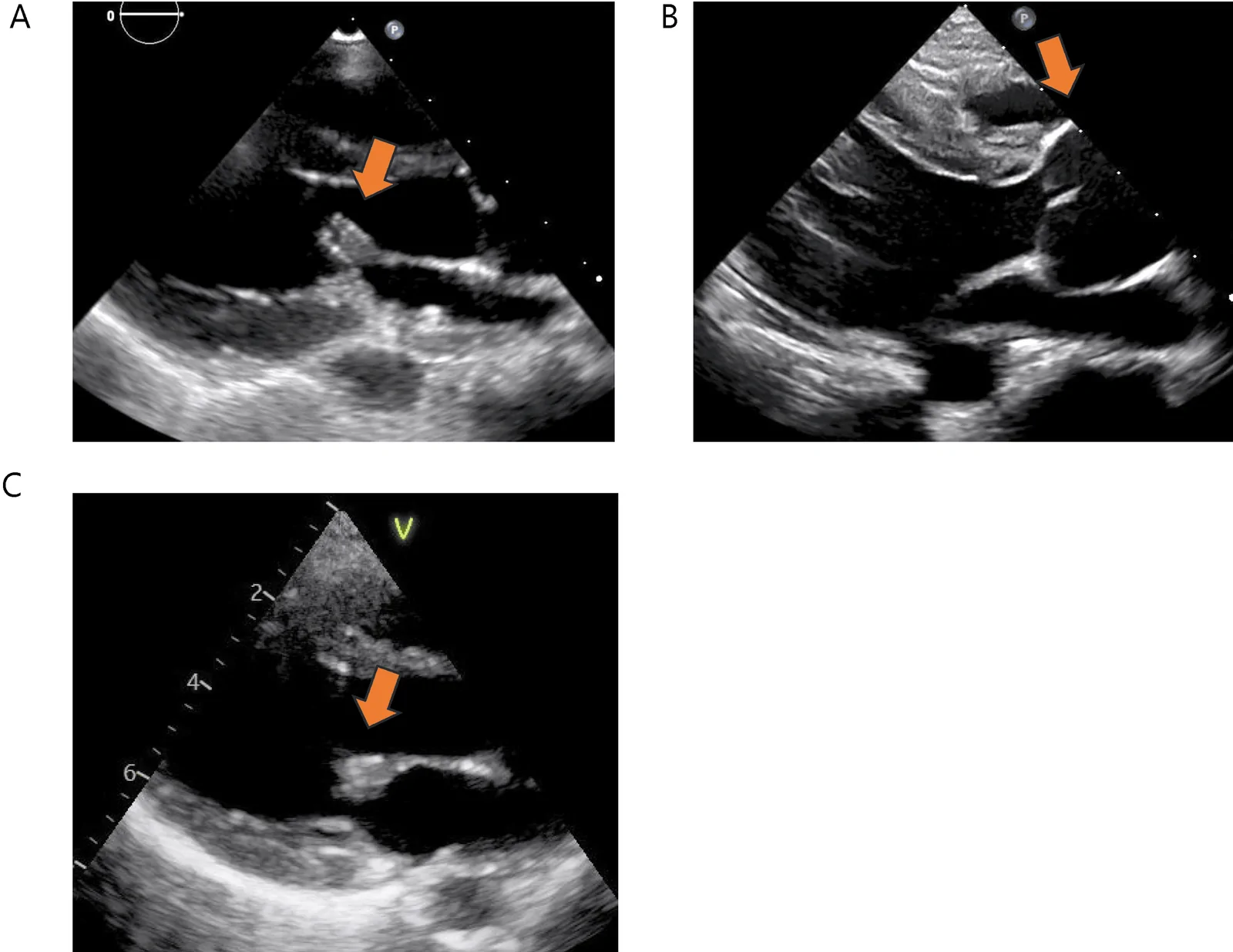
Echocardiography (A) Left parasternal long-axis view on admission shows an infective endocarditis (IE) vegetation-like structure on the anterior leaflet of the mitral valve. (B) Left parasternal long-axis view on admission also shows enlargement of the aortic sinus of Valsalva. (C) IE vegetation-like structure was recorded in a previous echocardiographic evaluation before the onset of this infection.

Transthoracic echocardiography showed moderate mitral regurgitation directed toward the posterior left atrium, trivial aortic regurgitation, and an IE vegetation-like structure on the anterior mitral leaflet with prolapse (Figure 2A and Video 1).

**Video 1:** Echocardiography on admission The left parasternal long-axis view shows an infective endocarditis (IE) vegetation-like structure on the anterior leaflet of the mitral valve.

The lesion was attached to the tip of the anterior mitral leaflet, measured approximately 10.2 × 6.8 mm, and demonstrated limited independent oscillatory motion. The mitral valve diameter was 38.4 mm (Z-score, +3.3). The sinus of Valsalva was enlarged (Figure 2B), with an aortic annulus diameter of 17.2 mm (Z-score, +1.14) and sinus of Valsalva diameter of 28.1 mm (Z-score, +3.59); Z-scores were calculated using "parameter Z" [8]. Although rare, IE due to *M. pneumoniae *was considered, and treatment with ceftriaxone, sulbactam/ampicillin, and clarithromycin was initiated. By the third hospital day, the patient's fever had resolved, inflammatory markers had improved, and chest radiographic findings showed continued improvement (Figure 1B). However, the vegetation-like structure on the anterior mitral leaflet remained unchanged.

Three sets of blood cultures were obtained: at the referring hospital before transfer, at admission to our institution before antibiotic administration, and again on hospital Day 8. All cultures remained negative throughout the clinical course. Review of echocardiographic images obtained approximately one year before the current admission at another institution during evaluation of an incidental heart murmur detected at a school health examination, while the patient was afebrile (Figure 2C and Video 2), demonstrated a similar vegetation-like structure. Consequently, antimicrobial therapy was discontinued on Day 13 of the treatment, and no recurrence of fever or progression of echocardiographic findings was observed during 12 months of follow-up.

**Video 2:** Echocardiography approximately one year before the current admission Infective endocarditis (IE) vegetation-like structure was recorded in a previous echocardiographic evaluation before the onset of infection.

At the time of the initial evaluation, the patient provisionally fulfilled one major criterion (an echocardiographic vegetation-like lesion) and one minor criterion (fever ≥38°C), corresponding to possible infective endocarditis according to the modified Duke criteria. However, subsequent clinical evaluation demonstrated that the mitral valve lesion represented noninfective myxomatous degeneration rather than true vegetation. Transesophageal echocardiography or cardiac computed tomography/magnetic resonance imaging was not performed because the patient remained clinically stable, there were no findings suggestive of complicated infective endocarditis, and subsequent review of a previous echocardiographic examination demonstrated that the lesion had been present for approximately one year before the current illness. Therefore, the final diagnosis did not fulfill the criteria for definite infective endocarditis.

Given the dilation of the sinus of Valsalva, a connective tissue disorder was suspected. Genetic testing performed at the Kazusa DNA Research Institute (Chiba, Japan) identified a heterozygous missense variant in FBN1 (NM_000138.5:c.7864T>A, p.Cys2622Ser), which was classified as likely pathogenic according to the American College of Medical Genetics and Genomics (ACMG) criteria [9]. The mitral leaflet abnormality was ultimately attributed to myxomatous degeneration, a known cardiac manifestation of Marfan syndrome. Ophthalmologic and orthopedic evaluations revealed no ectopia lentis or systemic skeletal features fulfilling the revised Ghent criteria [10]. Following the diagnosis of Marfan syndrome, the detailed family history was re-evaluated. There was no history of sudden cardiac death, aortic dissection, or markedly tall stature among first-degree relatives.

## Discussion

In this case, IE was initially considered in the differential diagnosis owing to the presence of fever, a heart murmur, and an IE vegetation-like structure identified on the mitral valve apex. However, the final diagnosis of myxomatous degeneration characteristic of Marfan syndrome was established based on three key factors: 1) although antibiotic therapy alleviated the fever and other systemic symptoms, the IE vegetation-like structure on the mitral valve remained unchanged; 2) a retrospective review of previous echocardiographic images revealed that similar valvular abnormalities had been present during an afebrile period; and 3) genetic testing subsequently confirmed Marfan syndrome. Histopathological confirmation was not available because surgical intervention was not indicated. Therefore, the final diagnosis was based on the clinical course, serial echocardiographic findings, and genetic confirmation of Marfan syndrome.

This case illustrates the diagnostic challenge posed by *M. pneumoniae *infection, as a negative blood culture does not exclude *Mycoplasma*-associated IE. Conventional blood culture systems are unsuitable for detecting *Mycoplasma *species in the bloodstream. Previous reports have documented *M. pneumoniae*-induced IE in cases where blood was cultured using specialized media or where clinical improvement occurred following doxycycline therapy despite persistently negative blood cultures [3]. A definitive diagnosis can be achieved through polymerase chain reaction detection of *M. pneumoniae *in the blood or isolation using specialized culture techniques; however, these methods were not used in the present case. In patients with culture-negative vegetation-like lesions, prolonged antibiotic therapy should be reconsidered when serial blood cultures remain negative, the clinical condition is stable, and integrated clinical assessment suggests a chronic noninfective process rather than active infective endocarditis. In our patient, antibiotic therapy was discontinued on hospital Day 13 after persistently negative blood cultures, clinical improvement, and retrospective review of prior echocardiographic studies demonstrating a similar longstanding lesion.

Mitral valve apex myxomatous (myxoma-like) degeneration with or without mitral regurgitation has been reported in patients with Marfan syndrome [11]. Marfan syndrome is associated with dysregulated TGF-β signaling [12], which may contribute to the development of myxomatous changes of the mitral valve [13]. These structural abnormalities lead to leaflet redundancy, prolapse, and irregular valvular thickening or protruding tissue, which may mimic vegetations on echocardiography. Myxomatous changes associated with Marfan syndrome typically do not undergo spontaneous regression. In the present case, the lesion detected during a febrile condition complicated the diagnosis. Moreover, typical phenotypic features of Marfan syndrome, such as tall stature, long extremities, arachnodactyly, and spinal deformities [10], were absent in this case. Echocardiography performed at another hospital revealed aortic root dilatation and redundancy of both the aortic and mitral valves, consistent with connective tissue disease, although myxomatous degeneration of the mitral valve was not recognized at that time. Although the precise frequency is unknown, myxomatous valvular changes can occasionally resemble IE vegetations, making echocardiographic differentiation challenging, particularly in patients without characteristic phenotypic features of Marfan syndrome. Genetic testing should be considered even in children without overt phenotypic features when unexplained valvular abnormalities, myxomatous valvular changes, or aortic root dilatation raise suspicion for an underlying connective tissue disorder. In the present case, the diagnosis of Marfan syndrome was supported by a pathogenic FBN1 variant together with aortic root dilatation according to the revised Ghent criteria [10]. In patients with Marfan syndrome, regular echocardiographic monitoring of mitral regurgitation and aortic root dimensions is necessary to detect progressive disease and determine the timing of potential surgical intervention.

Therefore, in patients, particularly pediatric patients, presenting with an IE vegetation-like structure on the mitral valve apex, it is crucial to consider differential diagnoses other than IE, including nonbacterial thrombotic endocarditis and Lambl's excrescences [14]. Additional imaging modalities, including transesophageal echocardiography or cardiac magnetic resonance imaging, may be helpful in selected cases. A limitation of this report is that no pathological examination of the mitral valve tissue was performed, and no imaging modalities other than echocardiography or radiography were utilized.

## Conclusions

This case emphasizes that vegetation-like lesions detected by echocardiography are not synonymous with infective endocarditis and may also occur in noninfective conditions, including myxomatous degeneration associated with Marfan syndrome. Although echocardiography remains the cornerstone for evaluating valvular lesions, echocardiographic findings alone may be insufficient to distinguish infective from noninfective lesions because both can present with similar vegetation-like appearances. Accurate diagnosis therefore requires comprehensive integration of the clinical course, microbiologic investigations, review of prior imaging, and consideration of underlying structural or connective tissue disorders. In the present case, retrospective review of a previous echocardiographic examination demonstrating that the lesion had remained unchanged for approximately one year, together with subsequent genetic testing, was essential in establishing the correct diagnosis. Even in children without characteristic phenotypic features, unexplained myxomatous valvular abnormalities or aortic root dilatation should prompt consideration of an underlying connective tissue disorder, for which genetic testing may provide important supportive diagnostic information.
